# A dynamic goal adapted task oriented dialogue agent

**DOI:** 10.1371/journal.pone.0249030

**Published:** 2021-04-01

**Authors:** Abhisek Tiwari, Tulika Saha, Sriparna Saha, Shubhashis Sengupta, Anutosh Maitra, Roshni Ramnani, Pushpak Bhattacharyya

**Affiliations:** 1 Dept. of Computer Science and Engineering, Indian Institute of Technology Patna, Patna, Bihar, India; 2 Accenture Labs, Banglore, Karnataka, India; National University of Singapore, SINGAPORE

## Abstract

**Purpose:**

Existing virtual agents (VAs) present in dialogue systems are either information retrieval based or static goal-driven. However, in real-world situations, end-users might not have a known and fixed goal beforehand for the task, i.e., they may upgrade/downgrade/update their goal components in real-time to maximize their utility values. Existing VAs are unable to handle such dynamic goal-oriented situations.

**Methodology:**

Due to the absence of any related dialogue dataset where such choice deviations are present, we have created a conversational dataset called Deviation adapted Virtual Agent(*DevVA*), with the manual annotation of its corresponding intents, slots, and sentiment labels. A Dynamic Goal Driven Dialogue Agent (DGDVA) has been developed by incorporating a Dynamic Goal Driven Module (GDM) on top of a deep reinforcement learning based dialogue manager. In the course of a conversation, the user sentiment provides grounded feedback about agent behavior, including goal serving action. User sentiment appears to be an appropriate indicator for goal discrepancy that guides the agent to complete the user’s desired task with gratification. The negative sentiment expressed by the user about an aspect of the provided choice is treated as a discrepancy that is being resolved by the GDM depending upon the observed discrepancy and current dialogue state. The goal update capability and the VA’s interactiveness trait enable end-users to accomplish their desired task satisfactorily.

**Findings:**

The obtained experimental results illustrate that DGDVA can handle dynamic goals with maximum user satisfaction and a significantly higher success rate. The interaction drives the user to decide its final goal through the latent specification of possible choices and information retrieved and provided by the dialogue agent. Through the experimental results (qualitative and quantitative), we firmly conclude that the proposed sentiment-aware VA adapts users’ dynamic behavior for its goal setting with substantial efficacy in terms of primary objective i.e., task success rate (0.88).

**Practical implications:**

In real world, it can be argued that many people do not have a predefined and fixed goal for tasks such as online shopping, movie booking & restaurant booking, etc. They tend to explore the available options first which are aligned with their minimum requirements and then decide one amongst them. The DGDVA provides maximum user satisfaction as it enables them to accomplish a dynamic goal that leads to additional utilities along with the essential ones.

**Originality:**

To the best of our knowledge, this is the first effort towards the development of *A Dynamic Goal Adapted Task-Oriented Dialogue Agent* that can serve user goals dynamically until the user is satisfied.

## 1 Introduction

### 1.1 Contextualization

In recent times, conversational artificial intelligence has become one of the prominent research areas because of its utility and efficacy [1]. Depending on the nature of the conversation, it can be divided into two categories namely: Task-oriented dialogue system [2–4], and Open-ended dialogue system [5, 6]. In Task/Goal Oriented Dialogue Systems, VAs intend to assist humans to accomplish a particular task efficiently. The user conveys a goal to the agent through a sequence of utterances. It also requests for a few necessary information required for the task completion, if the user has not conveyed this information, such as the number of people in case of movie ticket booking. The agent understands and serves the goal by performing an action (such as fetching an appropriate result) and completes the dialogue. In real world, the user may not be satisfied with the result presented by the agent and may want to update or change the goal. The user might want to interact with the agent about the information or result shown, trying to accommodate his/her feedback and finalize their task goal dynamically. DGDVA can help users maximize their utility through interaction and feedback and thus, make the agent more realistic and effective.

### 1.2 Relevance

Task-Oriented VAs intend to assist real users for a particular task. It is hard to assume that end-users will always have a predetermined goal. In real world, users may have some predefined goal components (minimum requirements) while also additionally trying to explore the capability of the virtual agent to maximize their utility. Thus, the proposed VA should be capable of dealing with such practical scenarios with maximum user satisfaction. The proposed methodology can be incorporated with any typical task-oriented dialogue system where end users may not have a predefined goal. The end users decide their exact task goal depending on their minimum predefined goal components and information retrieved through VA interactions. For example, in an interactive buying scenario, a buyer may deviate from their original goal when informed about the latent features of the shown item.

### 1.3 Research question

In task-oriented dialogue systems, agents complete the task by filling the necessary user task constraints (intent, slot) [7] and serve a goal matching the user specification. However, in real-world scenarios, users may not always have a predefined task goals, i.e., they might conclude their task depending on their minimum concerns and agent goal serving capability. So, the user goals are more likely to be dynamic rather than be driven by present constraints or specifications. To deal with such goal deviation, the dialogue agent should have the intelligence to identify goal deviations/discrepancies and update the goal appropriately. The agent should continue serving the user until and unless the user accomplishes his/her task adequately. To track whether the VA is leading the conversation in the user’s desired trajectory, user sentiment evolves as a reliable and feasible choice. It assists the VA in deciding whether to conclude the dialogue or to re-serve the user by incorporating his/her feedback in the previous goal. Hence, we have formulated user sentiment as an integral part of Goal driven module (Discrepancy Detector) that tracks discrepancy and triggers goal manager whenever required. Also, the reward/penalty awarded to the VA based on user sentiment explicates the appropriateness of its behavior at a given state. User sentiment provides grounded feedback about agent behavior [8]; thus, the VA can utilize it for learning an optimal dialogue policy.

### 1.4 Objective

This paper presents a dynamic goal adapted task-oriented dialogue agent that can adapt to goal deviations and serve user goals dynamically. The dialogue policy learning [9] task is formulated as a Partially Observable Markov Decision Process (POMDP) [10] with a unique state representation and a novel reward model. We created and annotated a dialogue corpus, *DevVA*, that contains conversation pertaining to user goal deviation. The agent utilizes user’s sentiment in dialogue policy learning as immediate feedback for identifying goal deviation/discrepancy and making the VA user-adaptive. The negative sentiment expressed by the user about an aspect of the provided choice is treated as a discrepancy that initiates a new goal. The incorporation of a dynamic goal driven module that tracks and updates user goals if any discrepancy occurs is the major difference with the traditional VA. User satisfaction is of utmost priority for any VA [11]. To successfully conclude a conversation, the user and the agent must collaboratively drive the interaction dynamically towards accomplishing a user satisfying goal.

The key contributions of the current work are as follows:

- We aim to develop a dialogue agent that can deal with dynamically changing user goals; Goal driven module (GDM) has been incorporated with the dialog manager (DM) to track user goals and update them accordingly.
- A large-scale dialogue dataset has been created containing conversations in the context of deviation of user goals for sales domain (Mobile Selling-Buying scenario). The dataset has been manually prepared and annotated with its corresponding intent, slot and sentiment labels. This dataset will be made publicly available for the research community.
- The proposed DM framework employs user sentiment for tracing goal discrepancy, and in case of discrepancy, it leads to the user’s dynamic goal without any interruption. Also, the additional sentiment-based immediate reward (SR) guides the VA to act more optimally as per user requisite and make it user-adaptive.
- The obtained experimental results and its post-investigation with real users (Human evaluation) show that the *DevVA* serves users’ dynamic goals with a significantly higher success rate in a reasonable dialogue turns.

### 1.5 Structure of the paper

Section 2 highlights the recent related work in the task oriented dialogue system, followed by the motivation of this work. Section 3 formulates the problem. Section 4 outlines the data creation and annotation process. Section 5 focuses on the proposed methodology. Section 6 describes the experimental setup. Section 7 presents the experimental result followed by a detailed analysis. The conclusion and future work are presented in section 8.

## 2 Background

### 2.1 Related work

A typical dialogue system comprises of three main components; namely a) Natural Language Understanding (NLU) [12] that converts natural language messages to structured data containing user intents and specific information called slots; b) Dialogue Manager (DM) [12] that selects one of the possible agent actions based on this structured information and dialogue history; c) Natural Language Generator (NLG) [13] that outputs the selected VA action in a user-understandable language. The fundamental task of a dialogue manager is to optimize dialogue policy, which decides the behavior of the dialogue system based on the given dialogue history. This dialogue optimization [9] problem can be viewed as a sequential decision making problem that can be solved efficiently through reinforcement learning [14] technique.

In these last few years, there has been an upsurge in research focused on deep learning based dialogue systems [15] due to the popularity of virtual agents both in industries and in social space. A VA can be trained primarily with two approaches: 1. Supervised Learning (Seq2Seq Model), 2. Reinforcement Learning. The first one is neural Sequence-to-Sequence (Seq2Seq) supervised approach [16], where an agent learns what to generate as a response given previous user utterances. The latter approach treats the dialogue manager as a Partially Observable Markov Decision Problem (POMDP) [17], which can be optimized by Reinforcement Learning (RL) technique. The key problem with the Seq2Seq approach is the requirement of a massive amount of dialogue corpus to ensure an optimal policy. On the other hand, data requirement in the latter approach is comparatively less as an RL based approach can be trained through simulated users. Additionally, RL agent needs to interact with the underlying environment but it is very costly as well as time-consuming to employ real users while training the agent from scratch. One feasible and well-accepted approach is to build a user simulator [18] based upon the problem and nature of the corpus.

#### 2.1.1 Seq2Seq based approach

In [19], authors proposed an end-to-end neural model that learns to generate end response directly through human-human conversational dialogue data. The task-oriented dialogue system for restaurant booking is proposed as a multi-task sequence learning problem with components of user input encoding, belief state tracking and agent response generation. In [20], authors presented a single seq2seq model with a two-stage copy mechanism to overcome the architectural complexity and fragility of the modular dialogue system. The first stage’s copy attention mechanism has been applied as input for encoding current belief state while the second assists in response generation from the belief state. An end to end memory network based dialogue agent has been proposed in [21] for training the agent to perform non-trivial tasks such as updating API and providing extra information. The dialogue agent can deal with slot updates through updating API. Our work is different in the way it provides flexibility to end-users for incorporating their suggestion/feedback in run time for accomplishing their desired task goal/dynamic goal.

#### 2.1.2 End-to-end task-oriented VA

In a conventional pipelined dialogue setting, downstream modules are significantly affected by previous modules and it becomes hard to diagnose and propagate loss to the erroneous module. An end to end neural dialogue system has been proposed in [22] to deal with drawbacks of a modularized task-oriented dialogue system. To deal with the reward sparsity problem of the early policy learning phase, a dialogue data augmentation (DDA) method that utilizes failed conversation for dialogue policy learning has been proposed in [23]. In [24], authors presented a simple and elegant two-stage technique to accelerate dialogue policy learning. The former one studies the effect of weight update frequency during exploration and exploitation and the later escalates the learning with a very limited size of mini-batch sampled from experience replay memory. In [25], authors have proposed a novel neural method for an efficient key-value retrieval required for Knowledge Base(KB) search in an end to end system. The model utilizes encoder-decoder architecture for context representation and is further augmented with an attention-based retrieval mechanism for efficient search from the underlying knowledge base. The author proposes a framework for consistent KB entity retrieval using a simple two-stage mechanism in [26]. The former retrieves the most relevant KB row depending upon dialogue history and the latter selects an appropriate attribute of the row using attention over attributes given decoded state representation. In [27], authors propose a global to local memory pointer (GLMP) network to deal with large and dynamic KBs which are hard to incorporate in the learning framework. The proposed model utilizes a global memory pointer generated depending upon dialogue context to filter external knowledge for relevant information. Then, it augments the slot values via the decoded local memory. As our underlying knowledge base contains comparatively fewer slots, a deterministic system performs nearly equivalent with significantly less complexity and computation. Although task-oriented dialogue agents attain remarkable success, they perform poorly to adopt a new domain with limited annotation. This work [28] addresses this issue and proposes a novel Dynamic Fusion Network(DF-Net). DF-Net exploits and utilizes the relevance between the target domain with each domain for improving the performance across all domains, including the target domain.

#### 2.1.3 Sentiment aware and persuasion based VA

RL based dialogue agents learn through rewards received from an environment in response to each action, so designing an appropriate reward model is very crucial and sensitive for any RL based dialogue system [29]. User sentiment can be treated as an explicit and grounded user feedback towards the agent’s behavior; henceforth, it can be utilized in the dialogue policy learning process to assist end-users in a more appropriate and personalized manner. In [30], authors have proposed a Hierarchical Reinforcement Learning (HRL) based agent for multi-intent dialogue setting. They have shown that sentiment reward, in addition to task-based reward, leads to a more efficient dialogue policy that ensures successful task completion with maximum user satisfaction. In [31], authors have proposed a multi-tasking model for dialogue act and sentiment classification on a new corpus extracted from Mastodon social network. The result reveals a correlation between these two tasks that can be utilized for transfer learning. A Deep Co-Interactive Relation Network (DCR-Net) has been proposed in [32], that emphasizes the mutual information between dialogue act and sentiment classification task. The proposed joint model’s obtained results outperform unified baseline models by a margin of 3-12%, which signifies strong co-relation. In [33], authors have investigated and presented the role of dialogue context in utterance understanding, i.e., intent, dialogue act, and emotion identification. This paper employs different perturbations to distort dialogue context and studies its impact on multiple tasks. In [34] authors have incorporated user sentiment information in dialogue policy learning to make dialogue manager more effective and user-adaptive for a single intent conversation. They have shown that dialogue policy outperforms the baseline policy trained without sentiment information in terms of both success rate and dialogue length. In [35], authors presented a persuasive dialogue system focused on the personal traits of the end-user. The dialogue agent analyzes user traits with dialogue context and then selects one of the explicit persuasion strategies that suits best as per user profile.

#### 2.1.4 Goal driven agent

In [36], authors have proposed an approach to simulate navy training with goal driven agent (GDM) that can make reasonable changes in goal if it observes any discrepancy. In [37], authors combined GDM with case-based reasoning (CBR) [38] as GDM requires substantial domain knowledge for goal reasoning. The proposed work is the first step towards incorporating GDM with a dialogue system so that the agent can adapt to dynamic goals for accomplishing a user task with maximum gratification. None of the existing dialogue agents [13, 20, 39–41] can assist user effectively to decide their goal dynamically as they have not been developed with this pragmatic concern.

### 2.2 Motivation

The primary task of any goal-oriented dialogue agent is to accomplish a user’s task with utmost user satisfaction. Existing virtual agents assume that the end-user will always have a predefined task goal, which will be served after filling the corresponding intent and slots. However, in real-life, users do not always have a generic pre-known task goal, i.e., they determine their precise task goal dynamically based on their utility value and the agent’s serving capability. They may upgrade/downgrade/update their goal components in real-time to maximize their utility value. This assumption emphasizes the gap between real human assistance and virtual agent assistance, where users have a flexible goal to maximize their utility. Existing task-oriented dialogue agents fail to adapt to user’s dynamic behavior and results in either unsuccessful dialogue or unsatisfied end user. To deal with this scenario, the presented VAs in the literature may completely flatter in such goal deviations.

**Fig 1:**
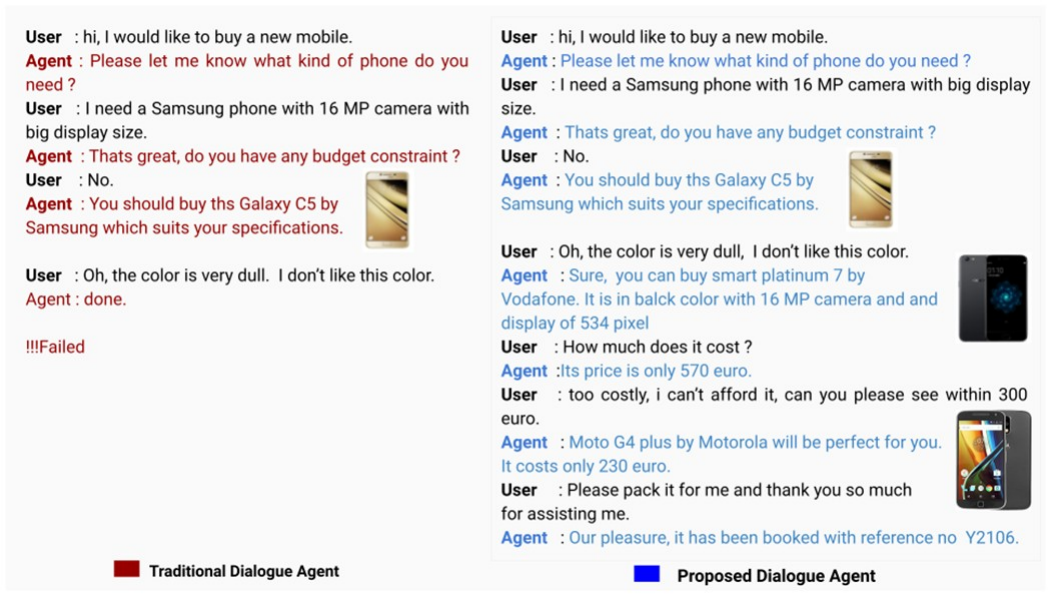
Performance of a traditional dialogue agent and our proposed agent in dynamic goal setting scenario.

Motivated by the inability of existing VAs, the proposed work aims to develop an advanced and dynamic goal-adapted dialogue manager for alleviating such scenarios. The following example (Fig 1) illustrates the objective and need of the proposed work. The proposed method can be incorporated with any generic task-oriented dialogue agent that can elevate them to deal with a dynamic and flexible goal effectively.

## 3 Problem formulation

**Fig 2:**
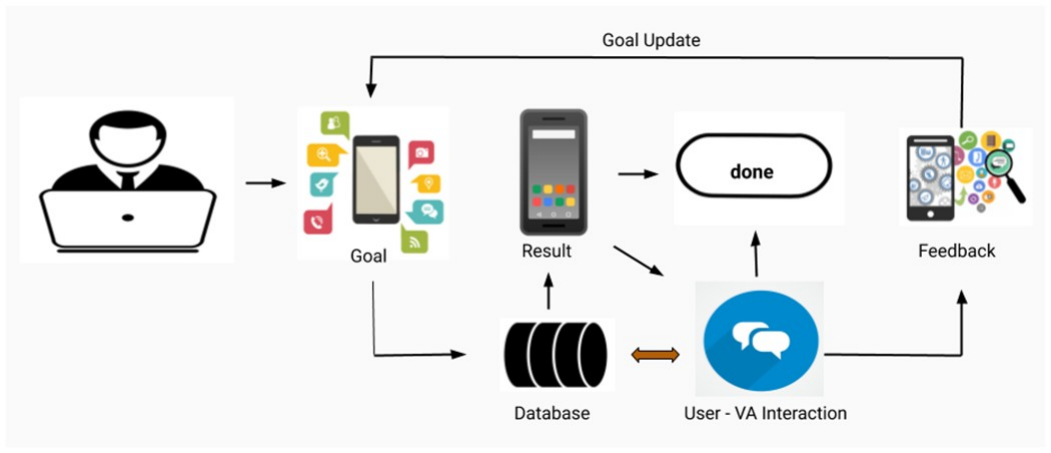
Dynamic Goal Driven Virtual Agent (DGDVA).

In real world, it can be argued that many people do not have rigid or fixed goals while conversing with a VA for a task such as buying a mobile phone or planning and booking for a vacation. User may change their goal depending on the availability of further information (slots) during an interaction. From Fig 2, it can be observed that users propose his/her initial goal, then after retrieving or knowing implicit features of shown result by the VA, users update his/her goal, which will be further served by the DGDVA.

Consider a situation where a user, (U) wants to buy a phone with “*front camera* = 13 MP” and “*battery capacity* ≥ 3000 mAh” (say). He/she starts a conversation with a sales agent (S) for the task. U begins with a salutation and tells his/her concerned task. Then, S requests some user informable slots or task’s features in general. U informs requested slots and his/her pre-decided goal components (“*front camera* = 13 MP, battery capacity ≥ 3000 mAh”). S performs slot-filling and shows a phone matching with the specified features. The user queries the phone’s price, S informs the same. Now, the user feels that the cost is out of his/her budget and expresses it to the agent. The agent recognizes the goal shift and updates the past goal based on the provided feedback. Then, the agent presents a new phone aligning with the user’s updated goal. Now, the user books the phone and concludes the conversation satisfactorily. A task (say Phone-Purchasing) has many other aspects (“price”, “color” etc.) than user predefined aspects (“front camera”, “battery” etc.). The disagreement in these latent feature values may lead to some deviation from the original choice.

The main objective of the proposed DGDVA is to learn an optimal dialogue policy π* for dynamic goal scenario using RL techniques. A dialogue policy *π* selects the most appropriate action for a current dialogue state, i.e., *π*(*s*, *gs*) → *a*. Here, *s*, *gs* represent current dialogue state and GDM state, respectively. A policy *π* will be an optimal policy(π*) if its cumulative episodic reward will always be greater than or equal to the cumulative episodic reward of any other policy. An optimal dialogue policy signifies that the agent behaves most accurately based on the dialogue context. Dialogue policy learning falls under the episodic RL problem where each episode consists of:

$$\begin{array}{c} \left(s_{0},gs_{0}\right)\Rightarrow \left[a_{0}\right]\left(s_{1},gs_{1},r_{0}\right)\Rightarrow \left[a_{1}\right]\left(s_{2},gs_{2},r_{1}\right)\Rightarrow \left[a_{2}\right]\left(s_{3},gs_{3},r_{2}\right),\ldots \ldots ,s_{n-1}\\ \Rightarrow \left[a_{n-1}\right]\left(s_{n},gs_{n},r_{n-1}\right) \end{array}$$

where *s*_*n*_ indicates current dialogue state, *gs*_*n*_ indicates current GDM state, *a*_*n*_ represents the action and *r*_*n*_ represents the reward for taking the action *a*_*n*_ leading to the transition into the state *s*_*n*+1_.

To make policy adaptable to goal shifts/deviations, goal driven module (GDM) tracks goal discrepancies through current user sentiment and dialogue state. It updates a user’s goal if it finds any goal discrepancy to serve his/her desired goal. The agent senses the correct and incorrect actions via a reward/penalty, which is provided by the environment for each action. Thus, the objective of the VA is to select actions in a way that maximizes discounted future rewards. The VA picks up an optimal action at every time-step based on the current dialogue state and the learned policy, which can be expressed as follows:

$$\begin{array}{c} a=argmax_{a\in A}\pi \left(s;\theta ;sentiment\right) \end{array}$$ (1)

where *A* is the set of all agent’s actions. *θ* represents all the parameters of the function approximator of the RL model. The model takes current state (s, gs) and user sentiment obtained through NLU processing of current user utterance and dialogue history. It selects the most appropriate action from the dialogue policy (*π*). The selected action is presented to the user after converting it into user understandable form through the NLG module to curate an end to end dynamic goal driven system.

## 4 Dataset

To motivate and advance research in dialogue system where VAs have to deal with dynamic user goals, we introduce a new dataset named DevVA.

### 4.1 Data collection

Several benchmark conversational datasets were explored for the proposed setting which include *ATIS* [42], *MultiWoz* [43], *Ubuntu dialogue corpus* [44], *bAbi* [45], *cornell-movie corpus* [46], *Deal or not* [47] etc. However, none of these open-sourced datasets was of goal deviation nature, i.e., the conversation terminates when the VA serves a fixed goal by eliciting slots. The bAbi corpus contains some user non trivial actions such as re-informing some slot-values, but these updates are occurring before the agent’s goal serving action. Thus, these publicly available datasets cannot be used for the dialogue scenario where users change their minds after seeing the result and its features shown by the VA.

### 4.2 Data creation and annotation

We created a sample conversation dataset containing 100 dialogues, where the agent serves user goals dynamically to complete the dialogue successfully (as per our need and scenario). These samples contain conversations between a buyer (user) and an online seller (VA) of mobile phones. It is annotated for its corresponding intent, slot and sentiment of each utterance. Three graduate students in English linguistics were then asked to create and annotate more samples for the dataset based on the provided sample conversations. The corpus contains conversations that emphasize on goal switch sensed through user sentiment.

### 4.3 DevVA dataset

**Fig 3:**
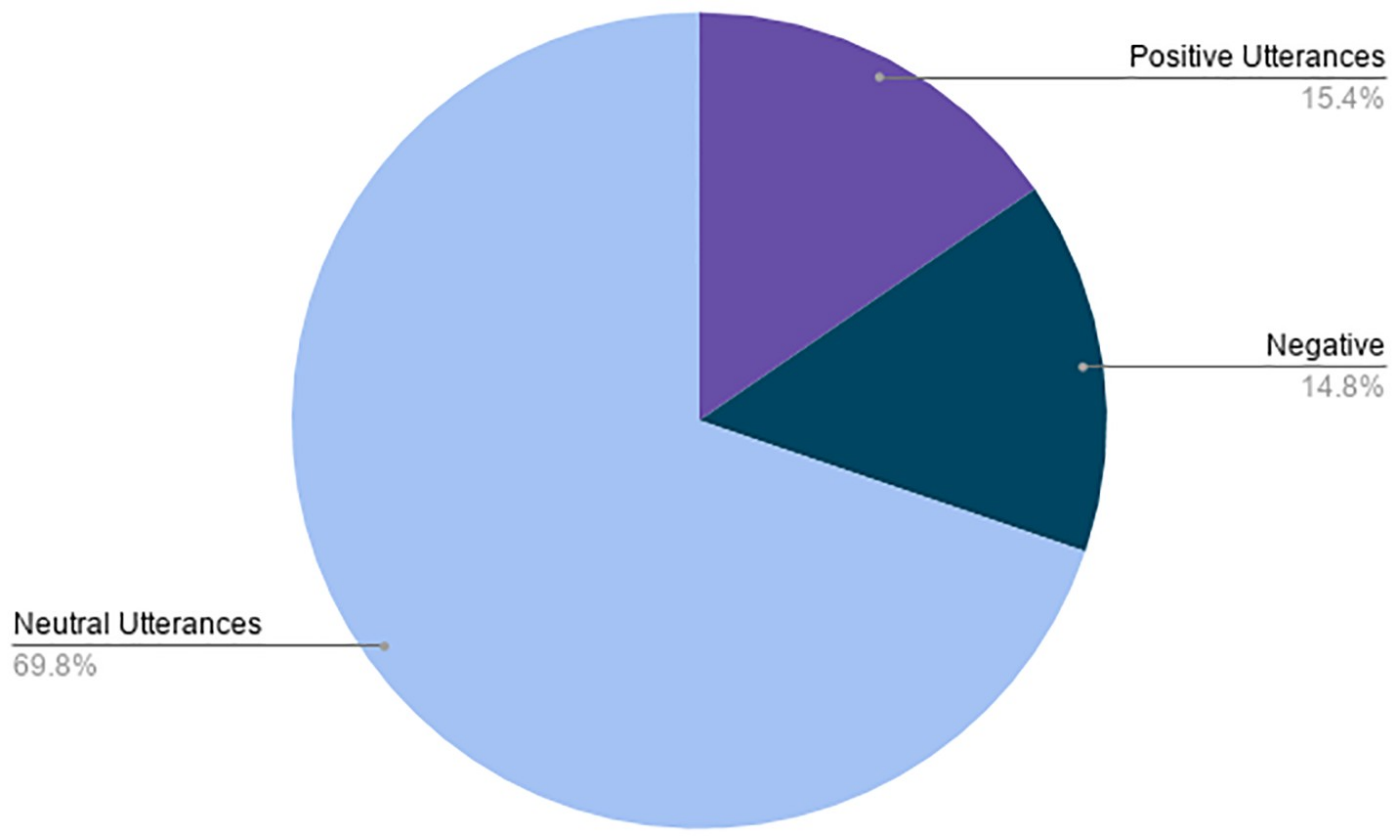
Sentiment class distribution of utterances of DevVA dataset.

The *DevVA* dataset now contains dialogues annotated with the corresponding labels for intent, slot, user action and sentiment. User sentiment has been categorized as *positive, negative and neutral*. A subset of the GSMArena [48] mobile database consisting of 2697 samples has been used as the knowledge base for creating conversations. The metadata and dialogue samples from the developed dataset are shown in Tables 1–4 respectively. To measure mutual agreement among annotators, we have calculated kappa coefficient (k), which was found to be 0.81. The sentiment class distribution of the dataset is shown in Fig 3.

**Table 1:** Statistics of DevVA dataset.

Entries	Values
Total No. of dialogues	1000
Total Utterances	8335
Average Dialogue length	8
No. of Slots	18

**Table 2:** Sample dialogue conversation 1.

User	Agent
Hi, I am in search of a good phone	hello, any specific phone do you need?
It should have big display and high memory	Is it all that you need?
Yes, and color should be black.	you can buy F3 by Oppo which has internal Ram of 64 GB and display size of 401 pixel.
What is its Primary Camera?	It is 13MP
Oh, but 13 MP is less, I take many pictures. show something with at least 15 MP.	Okay then V5 Plus by Vivo is perfect for you.
What is its weight	It is 184 gms.
Okay, I like this phone. Please Pack it for me.	Good choice, I am booking it for you.

**Table 3:** Sample dialogue conversation 2.

User	Agent
hi,can you help me in choosing a good phone?	Yeah Sure, What kind of phone are you looking?
It should be 2016 or latest released phone with 6 GB or more ram	Do you need any other features?
No, this is all my minimum requirements	you can buy M2017 by Gionee which has 6 GB Ram and it is released in 2016.
What is its Cost?	Its cost is 1150 euro.
Oh it is very costly, Is there anything below 500 euro?	You can buy 3T by OnePlus which costs only 440 euro.
What is its color?	The color of the phone is gunmetal.
But, my favorite color is black only	Then, you should take M6s Plus by Gionee.
Nice, Please Pack it for me.	Yeah, I am booking it for you.

**Table 4:** Sample dialogue conversation 3.

User	Agent
Hi, I want to buy a new phone	Sure, please let me know what kind of phone do you need?
It should be an Acer phone with high battery capacity.	Is it all your requirements?
I watch movie regularly, so display should be big.	Great, you should take Liquid Z6 that matches with your choice. It has a 4080 mah battery capacity and 401 inch display.
What are other features?	This phone contains 3 GB Ram, 13 MP camera …. 32 GB internal memory.
Nice, does it contain single slot for sim?	Yes, there is single slot for sim.
No, it is not sufficient for me. I need at least dual sim phone.	No issue, you can have Liquid Zade Primo which has two sim slots.
I have not heard its name, is it a latest phone?	This phone has been released in 2015
it is an old phone, see some latest one of 2016	Yes, you can take Iconic Talk S by Acer that will be perfect for you. It is released in 2016 only.
Yes please book it or me.	Sure, it has been booked for you.
Thanks buddy	You are most welcome. Hope you will enjoy this phone.

### 4.4 Qualitative analysis

- **Role of dynamic goal**: In real-life scenario, it is very usual not to have a rigid and predefined goal for a task. Users may diverge from their proposed goal for maximizing their utility value. In Table 2, user proposes his/her goal (*big display size and high memory capacity*). The VA serves the goal by showing a phone matching with the specified constraints. Most of the existing VAs terminate the conversation once they serve the proposed goal. But here, the user interacts with the VA to know other aspects of the phone and finds some unsuited feature (discrepancy). He/she provides feedback on the conflicting goal (*Camera Quality*) component, the VA re-serves the user through accommodating his/her suggestion into the previous goal. If it would be a typical VA, it may not be able to accommodate users’ feedback. Thus, it could lead to dialogue end without complete user satisfaction.
- **Role of sentiment**: Sentiment plays a vital role in identifying goal deviation/discrepancy as it is the only way of getting actual feedback regarding the agent’s served goal. In the provided sample (Table 3), the user expresses *negative* sentiment towards the *price* aspect of the proposed phone when he/she gets to know its cost. The Discrepancy module recognizes the negative sentiment as a discrepancy point and triggers the goal manager depending on user action. The goal manager updates the previous goal by incorporating the user’s feedback (*cost < 500*). The GDA Signal (discrepancy and updated goal) along with the current dialogue state are passed to the policy tracker. Next, the VA shows a phone matching with the updated goal. The user expresses his/her concern for the phone’s *color* and again the VA updates the former goal. Finally, the user’s goal is achieved on a positive note.

## 5 Materials and methods

**Fig 4:**
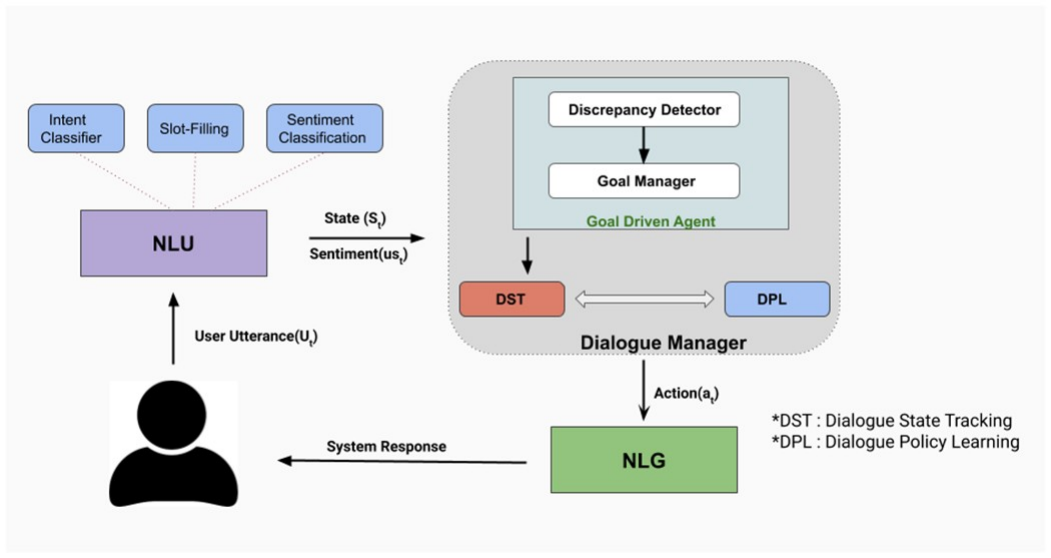
System architecture of the proposed methodology.

The fundamental component of any dialogue system is the dialogue manager, which should be capable enough to pick appropriate action depending on the conversational context. These sequences of appropriate actions help the end-user to complete his/her task successfully. We propose a dynamic goal-adapted dialogue manager comprising of the Goal Driven Dialogue Module (GDM), Dialogue State Tracker (DST) and Dialogue Policy Learner (DPL). GDM helps to detect discrepancies as well as provide the updated goal to the VA as per the observed discrepancy. Next, the VA chooses an action after applying the policy module on the current dialogue and GDA states. Fig 4 describes the control flow of the proposed dialogue system.

### 5.1 Goal Driven Module (GDM)

GDM is a goal reasoning model that revises its goal when any discrepancy occurs. The motivation of goal revision is to mitigate discrepancy’s effect on the agent’s ultimate goal, i.e., task completion. In a dialogue system, the agent’s fundamental concern is user’s task completion with maximum possible gratification. The major reason behind user annoyance (discrepancy here) is agent’s actions/served goals which are not aligned with the user goal. Hence, GDM has been incorporated with the DM to track discrepancies and update the goals accordingly.

The GDM comprises of two sub-modules named Discrepancy Detector and Goal Manager. In a dialogue system, user sentiment/feedback can be treated as a performance measure and thus a guiding parameter for policy learning. Our discrepancy detector detects discrepancy (General discrepancy or Goal discrepancy) based on the current user sentiment and its utterance. Then, it passes the discrepancy information to the next module, i.e., Goal manager. The explanation in terms of current user action, dialogue state and user sentiment is passed to the Goal Manager for tracking the goal and updating it. This module outputs discrepancy and its corresponding updated goal.

- **Discrepancy detector**: A reward for each DM action can be an intrinsic way of learning dialogue policy. There is another crucial feedback factor, i.e., user sentiment, which can be used for modeling dialogue policy learning process. Here, the discrepancy stage refers to the situation when a user expresses his/her feedback with negative sentiment towards the agent’s action.
  $$\begin{array}{c} D=\{\begin{array}{cc} 1, & \text{if}\;argmax\left(s_{t}\right)=0\;\text{and}\;user\_intent=1\;(\text{Goal}\;\text{discrepancy})\\ -1, & \text{if}\;argmax\left(s_{t}\right)=0\;\text{and}\;user\_intent\neq 1\;(\text{General}\;\text{discrepancy})\\ 0, & \text{Otherwise}\;\;(\text{No}\;\text{discrepancy}) \end{array} \end{array}$$ (2)
  where *D* denotes discrepancy information, (argmax(*s*_*t*_) = 0) signifies negative user sentiment and (user_intent = 1) indicates feedback as user intent.
- **Goal manager**: The main task of the goal manager is to formulate a new goal, *G*_*t*_ based on the previous goal, current user feedback and dialogue state (S_*t*−1_) in case of goal discrepancy. It takes discrepancy information, sentiment score, current user utterance and dialogue state as input and outputs GDM state/signal that contains discrepancy information, current goal and the sentiment score. The goal is updated through a deterministic function that can be represented as follows:
  $$\begin{array}{c} G_{t}=G_{t-1}\cup \left(U_{t}|D_{t},ss_{t},S_{t-1}\right) \end{array}$$ (3)
  $$\begin{array}{c} GDMState_{t}=\left[D_{t},ss_{t},G_{t}\right] \end{array}$$ (4)
  Where G_*t*_, U_*t*_, D_*t*_, ss_*t*_ and S_*t*−1_ denote user goal, user action, discrepancy, sentiment score and dialogue state at time *t*, respectively. In case of general discrepancy, the goal will be the same (G_*t*_ = G_*t*−1_) and the sentiment score (negative feedback) will help the VA in sensing the consequences of its immediate previous action.

### 5.2 Dialogue State Tracker (DST)

Dialogue state is used to represent dialogue conversation at a given time. The dialogue state provides a context that helps the dialogue policy module to choose appropriate action on the given context. Dialogue state tracker [49] tracks dialogue state, i.e., updates dialogue state after each user or agent utterance that incorporates essential information which is conveyed through the utterance. It takes processed user input from NLU as input and updates the previous dialogue state with it to get the current dialogue state, i.e.,

$$\begin{array}{c} S_{t}=StateTracker\left(S_{t-1},U_{t}\right) \end{array}$$ (5)

Where S_*t*_, S_*t*−1_ and U_*t*_ represent current state, previous state, NLU processed current user utterance, respectively. Our dialogue state space contains key information such as previous user action, user sentiment, agent request slot, user request slot, dialogue turn, knowledge base status, reward, etc.

### 5.3 Dialogue Policy Learner (DPL)

Dialogue policy is an integral part of dialogue manager that estimates a probability distribution over action space, i.e.,

$$\begin{array}{c} a^{*}=\pi \left(a|S\right) \end{array}$$

where S is the current state and a* is the action estimated with maximum probability through policy *π*. In a dialogue system, the agent’s main task is to predict the most appropriate action for a given state. It gets feedback in terms of reward for the transition (S, a, *S*′) from the environment. This policy learning problem can be viewed as a reinforcement learning (RL) problem where the agent learns through trial and error approach to optimize a policy. There are mainly two categories of RL algorithms known as Value-based algorithms and Policy-based algorithms. In Value-based methods, a policy is implicitly optimized through optimizing Q Value function. Whereas in a Policy-based algorithm, a policy is optimized directly through maximizing objective, i.e., cumulative episodic reward. We have optimized the dynamic goal driven policy through Deep Q Network [50], Actor-Critic Method [51] and their variants that are explained below.

**Fig 5:**
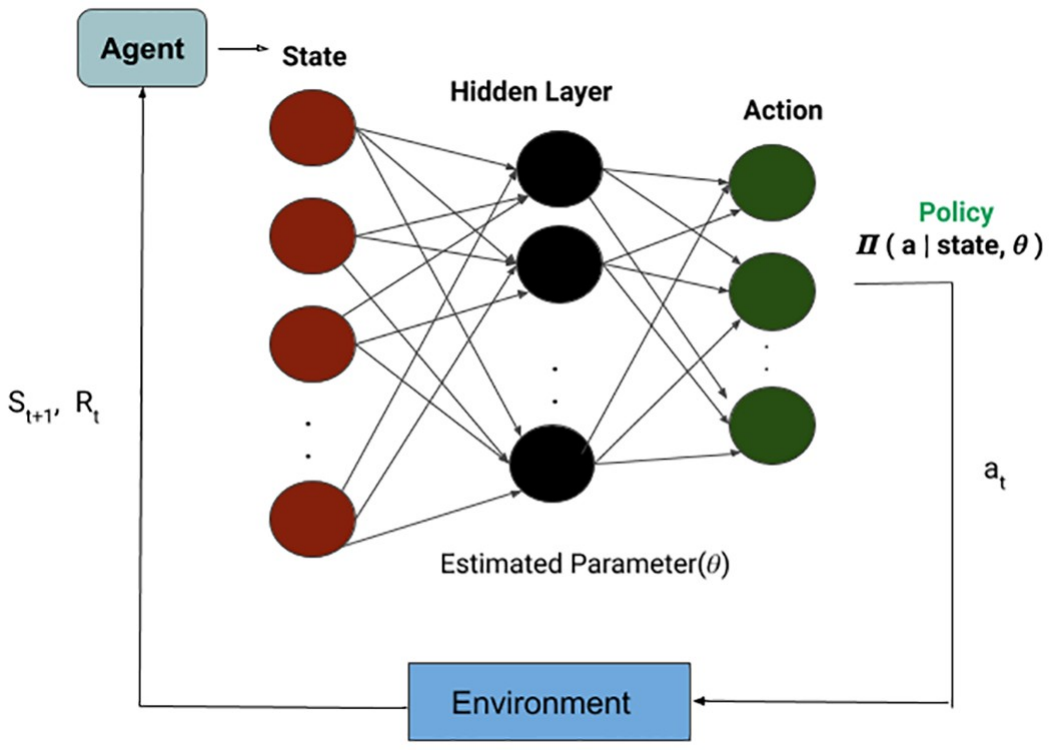
Dialogue policy optimization through a Deep Q Network(DQN).

**Fig 6:**
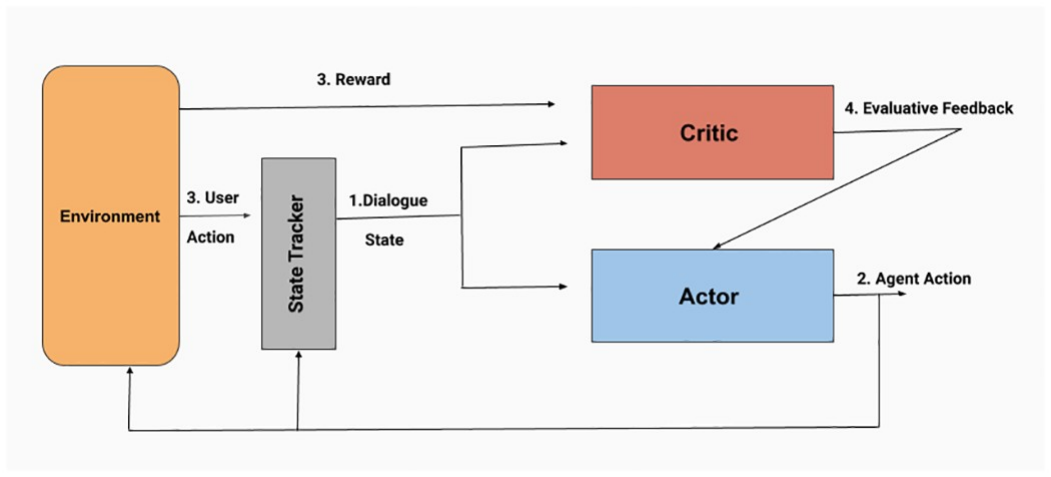
Actor Critic (AC) training architecture.

- **Deep Q Network (DQN)**: The DGDVA is modeled as a DQN [52] RL agent with input as dialogue state and GDM signal. The agent receives the current dialogue state and GDM input in structural form from the agenda-based user simulator [18]. The output of the network is a probability distribution over the action space. For each action, the agent gets feedback as a reward from the environment consisting of a user simulator and state tracker. The current dialogue state and GDM signal are used by the DQN agent to predict the next action. The control flow has been shown in Fig 5. It does so by computing Q(S,*a*_*i*_) where *i* ranges over all possible actions and then select the action which has the highest *Q* value. So, the problem reduces to approximating Q value function which can be optimized using the following Bellman equation:
  $$\begin{array}{c} Q\left(S,a\right)=E[r+\gamma \;\underset{a^{\prime }}{\text{max}}\;Q^{}\left(S^{\prime },a^{\prime }\right)] \end{array}$$ (6)
  where *S* is the current state, *a* is the action taken on state *S* which results in a new state *S*′ with a reward of *r*, *γ* is discount factor in the range of [0, 1] and *a*′ is the action on *S*′ that provides maximum reward.
  Dialogue policy optimization problem leads to Q function approximation that learns from temporal difference error. The error, temporal difference, is defined as:
  $$\begin{array}{c} L=\left[\left(r+r+\gamma \;\underset{a^{\prime }}{\text{max}}\;Q^{\prime (}S^{\prime },a^{\prime }\right)\right)-Q\left(S,a\right){)}^{2} \end{array}$$ (7)
  where *Q* is the prediction network and *Q*′ is the target network.
- **Actor Critic (AC)**: The Actor-Critic method [51] is an amalgamation of value-based RL algorithm and policy-based RL algorithm with the motivation of combined advantage. It consists of two networks: 1. Actor network that optimizes policy and predicts agent action based on the current state; 2. Critic Network evaluates the predicted action and provides feedback to the Actor Network. The training loop has been shown in Fig 6.
  In Policy Gradient method, the gradient of the objective function (J) is calculated with respect to the policy network parameter (*θ*) as follows:
  $$\begin{array}{c} \nabla_{\theta }J\left(\theta \right)=E[\nabla_{\theta }\left(log\left(\pi \left(s,a\right)\right)*Q_{\pi }\left(s,a\right)\right] \end{array}$$ (8)
  where *s*, *π*, *a* represent state, current policy and action taken by the agent in the state (s), respectively. *Q*_*π*_(*s*, *a*) is state-action value. The term ∇_*θ*_(*logπ*) indicates direction of *θ* in parameter space whereas the next term signifies whether the taken action *a* is good (if positive) or bad (if negative). The gradient ascent of the objective function encourages the agent to take more good action and less lousy action. However, it does not provide any insight on the goodness or badness of the taken action. It is one of the main motivations of the actor-critic method, where the critic network evaluates the goodness/badness of the action and provides corresponding feedback to the policy network (agent). The new gradient is defined as follows:
  $$\begin{array}{c} \delta =[\left(r+\gamma *V\left(s^{\prime }\right)\right)-V\left(s\right)] \end{array}$$ (9)
  $$\begin{array}{c} \nabla_{\theta }J\left(\theta \right)=E[\nabla_{\theta }\left(log\left(\pi \left(s,a\right)\right)*\delta \right] \end{array}$$ (10)
  In Eq 6, *δ* is the TD-error, *r* is the reward for taking action *a* in the current state *s*, which leads to a new state *s*′. V(.) signifies state value calculated through the critic network.

**Fig 7:**
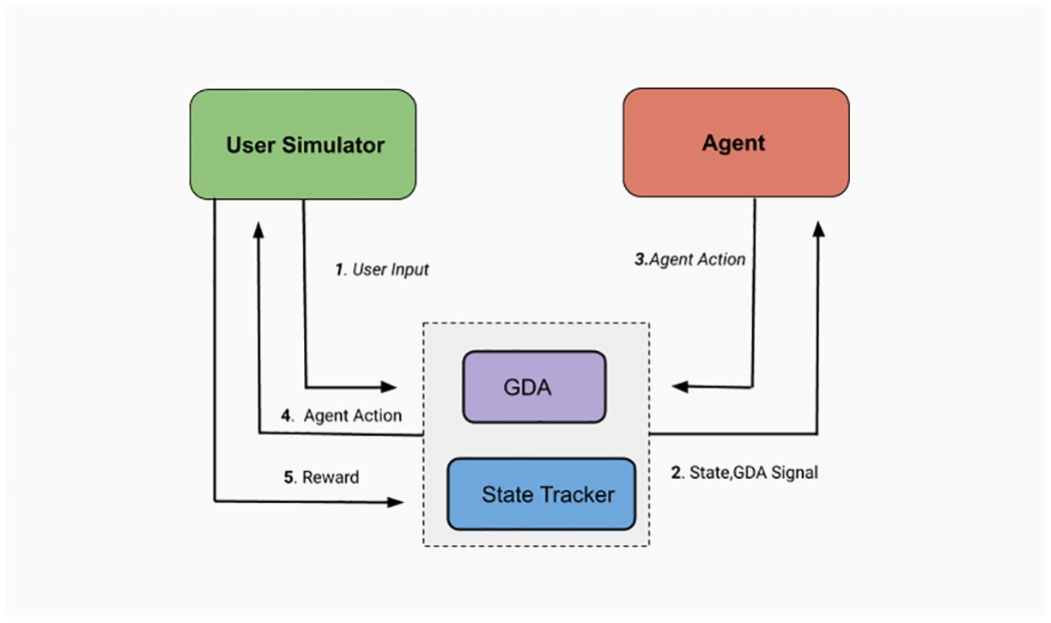
Training flow of the proposed RL based framework.

The training loop for the proposed dialogue system is shown in Fig 7. The pseudo environment takes user action based on the current user state and immediate agent action. The agent gets the updated dialogue state and GDM state as inputs and selects an action (a) as per the policy *π*. The agent learns about the action consequences (Q(S, a)) with the provided feedback in terms of reward/penalty for action, *a* from the user simulator.

### 5.4 State space

In our setting, the state space is a concatenated representation of the dialogue and GDM states. It is an array of 2n + 7s + N + 11 variables, where *n* is the number of intents, *s* is the number of slots and *N* is the maximum dialogue length limit i.e., 20 (here). The state contains context capturing variables such as current user action, previous agent action, agent inform slots, user informs slots, user request slots, which are represented as one hot embedding of intents (n: current user intent, n: previous agent intent) and slots. The dialogue turn’s one hot representation is also embedded in the state so that the agent can learn to complete dialogue in less number of turns as it gets the task success reward in terms of dialogue turns.

The proposed state is an extension of the Go-Bot Dialogue state [41]. It contains some new information such as sentiment score (ss), meta-data (user repetition, agent repetition) and GDM state. To capture the intensity of user sentiment, the obtained sentiment probability (s_*t*_) is scaled as a sentiment score in the range of [0, 1] as follows:

$$\begin{array}{c} ss=\{\begin{array}{cc} 1-p_{s}, & \text{if}\;argmax\left(s_{t}\right)=0\;(\text{tag}\;=\;\text{negative})\\ 0.5*p_{s}, & \text{if}\;argmax\left(s_{t}\right)=1\;(\text{tag}\;=\;\text{neutral})\\ p_{s}, & \text{Otherwise}\;\;(\text{tag}\;=\;\text{positive}) \end{array} \end{array}$$ (11)

### 5.5 Action space

The action space of the virtual agent consists of 7 categories having a total of 31 primitive actions. The categories are *specification* (e.g., *Sp* for asking specifications), *request* (e.g., BrandReq), *inform* (e.g., CostInform), *confirm* (e.g., Color(Red)), *result*, *done* and *salutation*. The actions are formulated after analyzing the problem, i.e., mobile selling environment and its feasible corner cases. Each agent action constitutes of intent/categories and its corresponding slots.

### 5.6 Reward model

We started with the following Task-oriented reward function (TR) shown in Eq 12 which mainly focuses on retrieving slot values. To motivate the agent for accomplishing user’s desired task in a minimum number of steps, the agent is penalized by a reward value of -1 for each action that leads to a non-terminal state.

$$\begin{array}{c} TR=\{\begin{array}{cc} +7*(MaxLenLimit-n) & \;\text{if}\;\text{success}\\ -2*MaxLenLimit & \;\text{if}\;\text{failure}\\ |LSlt^{\prime }-LSlt| & \;\text{if}\;(|LSlt^{\prime }-LSlt)|)\\ -1 & \;\text{otherwise}\; \end{array} \end{array}$$ (12)

Here, *TR*: Task Oriented Reward, *n*: No. of turns taken to complete, *LSlt*′: Length of Informed Slot in current state S’ and *LSlt*: Length of Slot list in previous state S.

$$\begin{array}{c} SR=\{\begin{array}{cc} 3*(s-1) & \;\text{if}\;s<0.5\;(\text{Negative}\;\text{User}\;\text{Sentiment})\\ s & \;\text{if}\;s=0.5\;(\text{Neutral}\;\text{User}\;\text{Sentiment})\\ 8*(s-0.5) & \;\text{otherwise}\;(\text{Positive}\;\text{User}\;\text{Sentiment}) \end{array} \end{array}$$ (13)

Here, *s* = Sentiment Score, *SR* = Sentiment based Reward.

With only TR, it was observed that the taken actions had some redundancies like repetitions, requests from a user that triggered unrelated requests from the agent and so forth. Repetition is a common issue with any dialogue manager and it may also cause irritating loops in the conversation. To avoid inappropriate actions from the agent’s side, we incorporated user sentiment information that acted as explicit feedback to the agent during the learning process. This sentiment-based reward (SR) shown in Eq 13 helps DM to understand whether it has picked an appropriate response related to the state of the dialogue. Thus, the final reward at each time-step is:

$$\begin{array}{c} Reward=TR+SR \end{array}$$ (14)

The proposed agent with combined reward function did not exhibit any loop as it was highly penalized for such actions during training. However, if the user is repeating something, it implies that the agent has not chosen the appropriate action against initial intent; it would trigger negative user sentiment for which the agent would be penalized. Similarly, the scenario where the agent generates an unrelated question in response to a user query also attracts a negative reward. The scalar numbers present in the reward functions are chosen empirically. The reward model utilizes the transformed sentiment score (ss) to reward/penalize the agent within the appropriate direction with higher confidence.

### 5.7 Case study

Lets say, s_*t*1_ = [0.6, 0.3, 0.1] and s_*t*2_ = [0.9,0.01,0.09], here both convey negative sentiment with ss_1_ = 0.4 * (1—0.6) and ss_2_ = 0.1 * (1—0.9). The penalties will be *r*_1_ = -1.8 * (- 0.6 * 3) and *r*_2_ = -2.7 * (-0.9 * 3). It might seem as a small difference but it matters significantly in discounted reward calculation (long term reward) which is defined in Eq 4. Thus, it helps the agent in learning consequence of an action in a more better way.

**Algorithm 1** Proposed GDM Incorporated Dialogue Policy Learning Algorithm with DQN(A) and ACM(B)

1: **Input**: User actions(*U*_[1:*t*]_), Agent’s previous actions (*A*_[1:*t*−1]_)

2: **Initialize**: Training episodes, Hyper Parameters, TrainingFreq, Previous State *s* with *U*_[1:*t*−1]_, *A*_[1:*t*−1]_, Experience Replay Memory(M) with WarmUp Transitions through Rule based Agent, Deep Q-Network (D^*θ*^) with experience replay memory M, Q state action function (Q(S,a)) with random weight *θ*, Target Q state action function ($\hat{Q}$(S,a)) with $\hat{\theta }=\theta$

3: **Output**: Agent action $({\text{A}}_{t})={\text{argmax}}_{a}\Pi_{DQN}^{*}\left(A|S,g\right)$     ⊳ Π*: Optimal Policy

**repeat**              **DQN** (*π*_*DQN*_)

  R

 **until**

4: ;

 eset environment, Dialogue state (S), GDM State (GS), done = false **repeat**

**repeat**

  g

 **until**

5: ;

 = GDM(*U*_*t*_, s, *S*_*t*−1_)        ⊳ s: user sentiment, g: GDM Status

6: S = getstate(*U*_*t*_, s, *S*_*t*−1_)        ⊳ S: Current State

7: a = argmax_a_ *π*_*DQN*:*θ*_ (A| S, g)        ⊳ A: Action Space

8: user action, reward = user(a)        ⊳ reward = TR + SR

9: $\text{done}=\{\begin{array}{ll} 1, & \text{if}\;\text{a}\;\text{is}\;\text{the}\;\text{terminal}\;\text{action}\\ 0, & \text{otherwise} \end{array}$

10: *S*′ = UpdateState(S, a, user action, reward, done)        ⊳*S*′: Next State

11: Append the experience tuple (S, a, *S*′ reward, done) to the experience replay memory(M)

12: S = *S*′

13: done

14: **if**(TrainingEpisode% TrainFreq)

15:  E = Sample random mini-batch of experiences from M        ⊳ E_*i*_ = (State, action, NextState, reward, done)

16:   ${\text{target}}_{i}=\{\begin{array}{ll} reward_{i}, & \text{if}\;{\text{done}}_{\text{i}}=1\\ reward_{i}+\gamma *{\text{max}}_{a^{\prime }}\hat{Q}({\text{NextState}}_{i},a^{\prime }) & \text{Otherwise} \end{array}$        ⊳, *γ*: Discount factor

17:  *θ*_*k*+1_ = *θ*_*k*_ − *α* * [(*Q*(*State*_*i*_, action) − target_*i*_)^2^]        ⊳ *α*: Learning rate

18:  $\theta =\hat{\theta }$

19: convergence        ⊳ Number of training episodes

                 **ACM** (*π*_*ACM*_)

1: **Input**: User actions(*U*_[1:*t*]_), Agent’s previous actions (*A*_[1:*t*−1]_)

2: **Initialize**: Training episodes, Hyper Parameters, TrainingFreq, Previous State *s* with *U*_[1:*t*−1]_, *A*_[1:*t*−1]_, Actor Network (AC^*θ*^), Critic Network (AC^*φ*^), Q state action function (Q(S,a)) with random weight *θ*, State value function (CriticValue(S)) with random weight *φ*

3: **Output**: Agent action $({\text{A}}_{t})={\text{argmax}}_{a}\Pi_{AC}^{*}\left(A|S,g\right)$        ⊳ Π*: Optimal Policy **repeat**

  R

 **until**

4: ;

 eset environment, Dialogue state (S), GDM State (GS), done = false **repeat**

  g

 **until**

5: ;

 = GDM(*U*_*t*_, s, *S*_*t*−1_)        ⊳ g: GDM Signal

6: S = getstate(*U*_*t*_, s,*S*_*t*−1_)        ⊳ S: Current State

7: a = argmax_*a*_ *π*_*AC*:*θ*_(A | S, g)        ⊳ A: Action Space

8: user action, reward = user(a)        ⊳ reward = TR + SR

9: $\text{done}=\{\begin{array}{ll} 1, & \text{if}\;\text{a}\;\text{is}\;\text{the}\;\text{terminal}\;\text{action}\\ 0, & \text{otherwise} \end{array}$

10: *S*′ = UpdateState(S, a, user action, reward, done)        ⊳ *S*′: Next State

11: *δ* = [*reward*(*S*, *a*) + *γ* * *CriticValue*_*φ*_(*S*′)] − *CriticValue*_*φ*_(*S*)

12: ∇_*θ*_ *J*(*θ*) = [∇_*θ*_(*log*(*π*(*A*|*S*)) * *δ*]

13: ∇_*φ*_ *J*(*φ*) = ∇_*φ*_ *δ*^2^

14: *θ* = *θ* + *α* * ∇_*θ*_ *J*(*θ*)        ⊳ *α*: Actor Learning rate

15: *φ* = *φ* + *β* * ∇_*φ*_ *J*(*φ*)        ⊳ *β*: Critic Learning rate

16: *S*′ = S

17: done

18: convergence        ⊳ Number of training episodes

## 6 Experimentation details

### 6.1 Training and testing

RL agent needs to interact with the underlying environment to perceive the consequences of the taken action. In our case, the underlying environment is end-users. However, interacting with real-user for training from scratch is a highly expensive choice. It might be a very tedious task for end-users and also may lead to a biased VA. The most feasible and economical solution is to initially train the VA with a user simulator and then extend it to the real corpus. Hence, we have developed a pseudo environment that mimics user behavior as per the domain. The pseudo environment, i.e., user simulator takes user action consisting of intent, slots and sentiment score as per the sampled goal and user state. The VA is first trained with the user simulator and later tested against the curated *DevVA* dataset. All the results reported below during testing are conducted with 30% of the total data.

### 6.2 NLU module

Natural language understanding is the initial and indispensable module of a dialogue system that converts user utterance to its schematic form [53]. The primary tasks of the incorporated NLU are Intent Classification (IC), Slot Filling (SF) and Sentiment Classification (SC). It processes the original user utterance through these sub-modules to provide its schematic which can be comprehensible for the next module of the pipeline, i.e., DM. We experimented and incorporated the pre-trained joint BERT model for intent and slot labeling [54], which is a state-of-the-art method for intent classification and slot tagging tasks. We have also experimented with two more state-of-the-art models for intent detection and slot labeling, namely joint capsule [55] and SF-ID [56].

**Fig 8:**
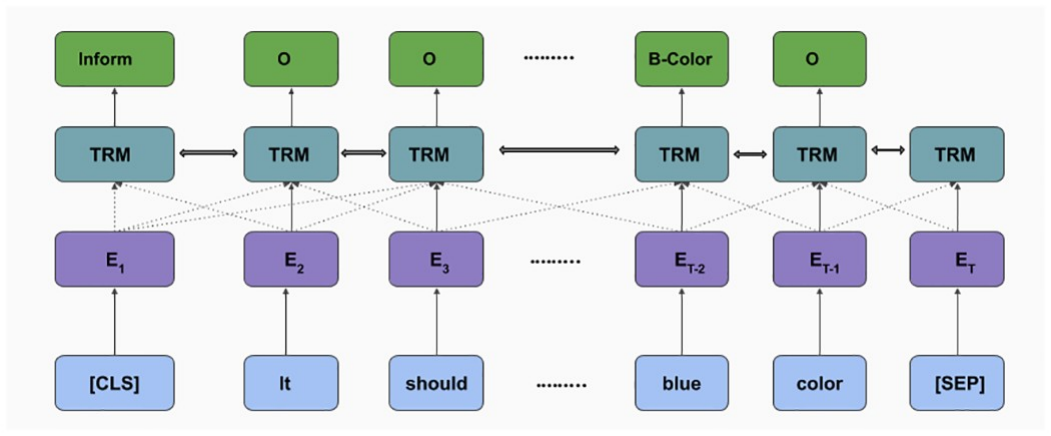
BERT for joint intent classification and slot filling.

- **Joint intent classification and slot filling module**: This module is responsible for intent classification and slot filling of user input (U_*t*_). It takes user response and predicts its intent and necessary information slots contained in it. We experimented with joint intent classification and slot filling models as these models capture inter-dependence between these two tasks and learn a better hidden representation that outperforms a baseline with two different Recurrent Neural networks (RNN).
- **BERT for joint intent classification and slot filling**: The BERT (Bidirectional Encoder Representations from Transformers) [54] is a multi-layer bidirectional Transformer [57] network that utilizes a concatenated representation of WordPiece embeddings [58], positional embeddings and the segment embedding for learning an efficient language hidden representation. The BERT model takes as input, x = *x*_1_, *x*_2_, *x*_3_……‥*x*_*T*_, appended with a special classification embedding (CLS) and [SEP] token as the first and last token. The embedded representation is passed to the next layer (Transformer network) that produces a hidden state, H = *h*_1_, *h*_2_, *h*_3_…‥*H*_*T*_, which is used for intent classification and slot filling as follows:
  $$\begin{array}{c} y^{i}=softmax\left(W^{i}h_{1}+b_{i}\right) \end{array}$$ (15)
  $$\begin{array}{c} y_{j}^{s}=softmax\left(W^{s}h^{j}+b^{s}\right),j=1,2,3\ldots .N \end{array}$$ (16)
  where y^i^, W^i^, h_1_ and b_*i*_ are predicted intent, weight matrix, special classification embedding (CLS) and bias matrix at time step *j*, respectively. ${\text{y}}_{J}^{S}$ indicates slot tag of ${\text{x}}_{j}^{th}$ input and *N* is the number of tokens in the input sequence. In Fig 8, an illustration has been shown of how the input data and the control flows in the model. To jointly train the model for intent classification and slot filling, the learning objective which needs to be maximized is defined as follows:
  $$\begin{array}{c} p\left(y^{i},y^{s}|x\right)=p\left(y^{i}|x\right)\Pi_{j=1}^{j=N}p\left(y_{j}^{s}|x\right) \end{array}$$ (17)
  This pre-trained model is fine-tuned with 80% of the total data (*DevVA*) and tested with remaining utterances.
- **Joint Slot Filling and Intent Detection via Capsule Neural networks(Joint SFIDCN)** [55]: In [55], authors proposed a capsule [59] based neural model which utilizes semantic hierarchy for joint modeling of intent detection and slot tagging task via a dynamic routing-by-agreement schema. It achieves the state-of-the-art performance on two publicly available corpora (ATIS and SNIPS).
- **A novel bi-directional interrelated model for joint Intent Detection and Slot Filling(SF-ID)** [56]: In [56], authors proposed a bi-directional interrelated model for joint slot filling and intent detection (SF-ID). The proposed SF-ID network is composed of slot filling (SF) and Intent detection (ID) modules, which utilizes intent information, slot information in slot filling task and intent detection task, respectively.
  The obtained results have been reported in Table 5.
- **Sentiment Classification (SC) module**: To identify the sentiment associated with a given utterance (U_*t*_), this module has been trained with the *DevVA* dataset.
  $$\begin{array}{c} s_{t}=SC\left(U_{t}\right) \end{array}$$ (18)
  where the input U_*t*_ is user utterance at t^th^ timestamp and s_*t*_ is the probability of each sentiment label, i.e., *positive, neutral and negative*. Experiments were performed with different models namely Pre trained BERT [60], Pre trained XLNet [61], GRU [62], LSTM [63], Bi-LSTM [64], Bi-LSTM + Attention similar to [65].
  XLNet [61] is an autoregressive language model that utilizes bi-directional contexts by maximizing the expected likelihood of overall permutations of the input sequence order. We have fine-tuned pre-trained XLNet for sequence classification (xlnet-base-cased) model with our dataset, *DevVA*. The Bi-LSTM model consists of an embedding layer, a hidden layer with 80 neurons and a softmax layer. Results are presented in Table 6. The performance of sentiment classification with XLNet is superior because of a more robust and better pre-training. We incorporated it as the sentiment classifier in our end-to-end setting.

**Table 5:** Results for intent classification and slot tagging tasks.

Model	Task	Accuracy(in %)	F1-Score
Joint BERT	Intent Classification	93.11	0.874
	Slot Tagging	87.39	0.866
Joint SFIDCN	Intent Classification	82.90	0.808
	Slot Tagging	85.18	0.827
SF-ID	Intent Classification	92.55	0.865
	Slot Tagging	85.04	0.839

**Table 6:** Results of different models for sentiment classification.

Model	Accuracy	F1-Score
GRU	94.25%	0.941
LSTM	94.31%	0.941
Bi-LSTM	94.61%	0.943
Bi-LSTM + Attention	95.02%	0.946
Pre trained BERT	91.37%	0.910
Pre trained XLNet	**96.68%**	**0.956**

### 6.3 NLG module

The NLG module randomly selects one of the templates from a predefined set of templates for each action before presenting the response to users. Once the VA picks an action followed by the knowledge base stage that fills required details (slot value/result), this final action is converted into a natural language form through this template based NLG module [66, 67].

### 6.4 Model architecture

- **DQN model**: The policy network consists of three layers: an input layer with a size of the concatenated representation of dialogue state space and GDM state, a hidden layer of 70 neurons and an output layer with a size equivalent to the length of agent action space. The training has been done on 50,000 dialogues. The final value of the hyper-parameters are as follows: learning rate (*α*) = 1e -3, discounted factor (*γ*) = 0.9, batch size = 32, experience replay size = 50,000, maximum dialogue length = 20. These hyperparameters are selected based on sensitivity analysis.
- **Actor critic model**: It consists of two neural networks: Actor that optimizes policy and Critic that estimates the value of a state. The Actor-network has three layers: an input layer with the size of the concatenated representation of dialogue state space and GDM state, a hidden layer of 200 neurons and an output layer with a size equivalent to the length of agent action space. The critic network has the configuration as the actor with one difference, i.e., its output layer has only one node that predicts the value of the given state. The final value of the hyper-parameters are as follows: actor learning rate (*α*) = 1e-3, critic learning rate (*β*) = 5e-3, discounted factor (*γ*) = 0.97, batch size = 32, experience replay size = 50,000, maximum dialogue length = 20. These hyperparameters are selected based on thorough sensitivity analysis.

## 7 Results and discussion

There are two ways to evaluate a virtual agent: automatic evaluation and manual evaluation. We have assessed the VA with both kinds of evaluation paradigms. Automatic dialogue evaluation is itself a challenging problem that is becoming an emerging research direction in the current dialogue community. The following three most popularly used automatic evaluation metrics [68–70] are utilized for quantitative evaluation of the proposed model:

1. **Success rate**: Dialogue will be completed successfully if and only if the VA serves user’s desired goal that follows user’s task specification and should answer all user queries within the maximum dialogue length limit (20 here).
2. **Average reward**: Average of reward values over total episodes.
3. **Average dialogue length**: Average of dialogue length over total episodes.
4. **Learning curve during training**: It shows how the VA is learning to optimize the objective, i.e., maximizing total reward over an episode. It signifies the appropriateness of the agent’s behavior over training episodes.

### 7.1 Comparison with the baselines

In order to establish the superiority of the proposed DM technique, it is compared with several other techniques which provide a space of fair comparison and analysis. The following models have been used as baselines:

- **Random agent**: Agent that takes a random action from the defined action space.
- **Rule agent**: It requests a fixed set of slots and attempts to predict goal from information received through interaction
- **Vanilla DQN**_*TR*_ **agent**: Only one Q network is used for both action selection and action evaluation. Reward model consists of task based reward only.
- **Vanilla DQN**_*TR*+*SR*_ **agent**: Vanilla DQN with both task and sentiment based rewards.
- **Actor Critic**_*TR*_: Actor Critic agent with solely task based reward.
- **Actor Critic**_*TR*+*SR*_: Actor Critic agent with both task and sentiment based rewards.
- ${\text{DDQN}}_{\text{TR}+\text{SR}}^{\text{B}}$ **Agent**: DDQN_*TR*+*SR*_ with BERT as sentiment classifier.

Results reported in Table 7 clearly establish that the proposed system performs much better compared to rule-based system, random agent and other baselines in terms of different evaluation metrics like *Success rate*, *Avg. Reward* and *Avg. Dialogue Length*.

**Table 7:** Results obtained by different dialogue agents.

Agent	Success rate	Avg. Reward	Avg. Dialogue Length
Random Agent	0.011	-232	15.25
Rule Agent	0.000	-127	11.00
AC_*TR*_ Agent	0.6615	36.11	11.32
AC_*TR*+*SR*_	0.6753	37.18	11.20
Vanilla DQN_*TR*_ Agent	0.8413	68.41	9.30
Vanilla DQN_*TR*+*SR*_ Agent	0.8573	70.82	8.91
DDQN_*TR*_ Agent	0.8693	73.02	8.68
${\text{DDQN}}_{TR+SR}^{B}$ Agent	0.8610	72.14	8.52
${\text{DDQN}}_{TR+SR}^{XL}$ Agent	**0.8904**	**79.86**	**8.38**

**Fig 9:**
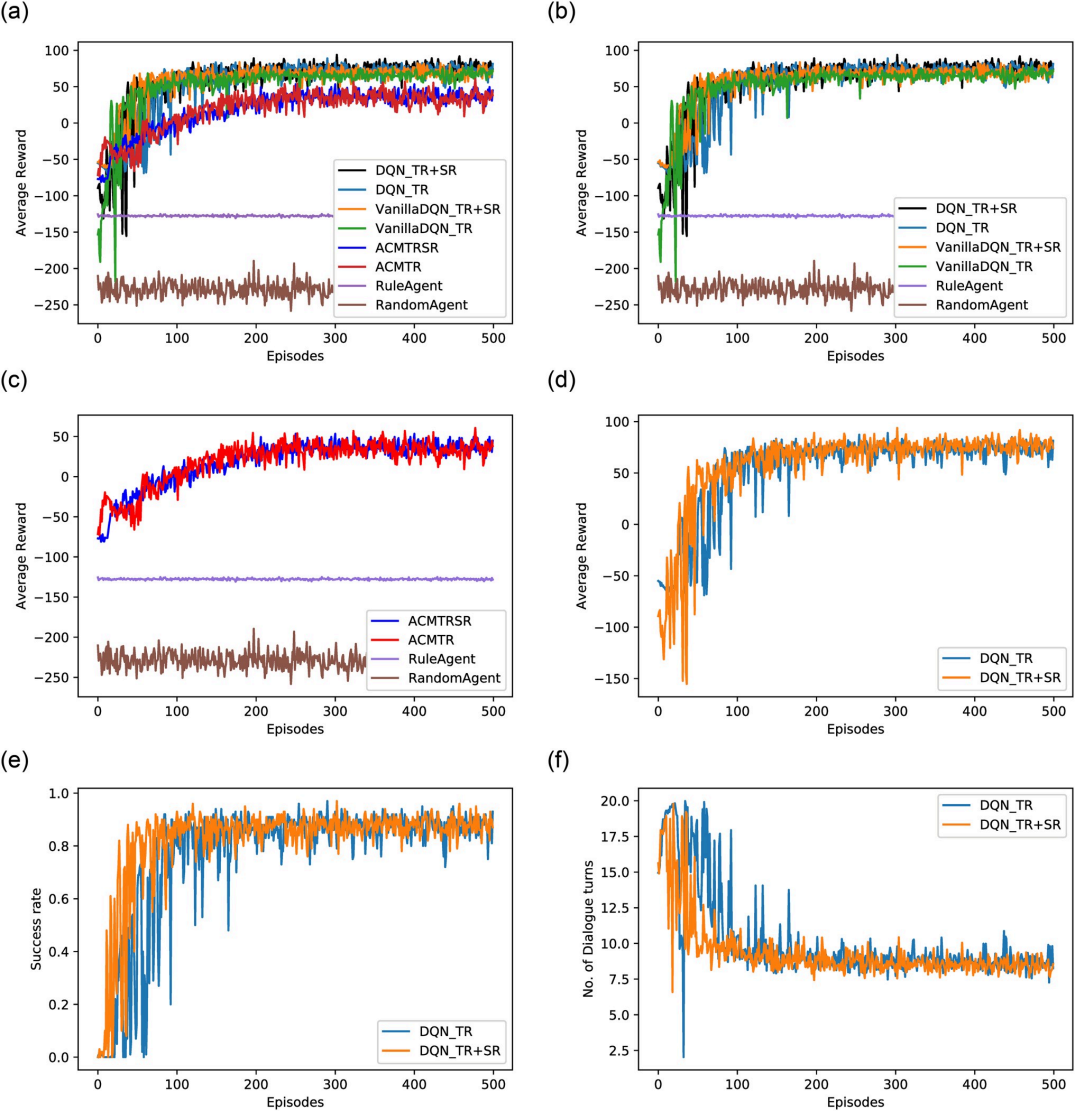
Learning Curves: (a) Learning graphs of different agents, b) Reward graph over episodes: Vanilla DQN and DDQN agents, c) Reward graph over episodes: Actor Critic, Rule agnet and Random agent, d) Reward graph over episodes: DDQN agents, (e) Success rate over episodes: DDQN agents, (f) Dialogue length over episodes: DDQN agents.

Fig 9a shows the learning of different agents during training. The random agent’s episodic reward does not improve over episodes as it takes action randomly without considering dialogue context, which leads to dialogue failure and a massive penalty. The rule-based agent’s episodic reward is constant over the episodes as it taking a fixed set of actions in all episodes. Also, its success rate is 0 (Table 7) because it only takes a fixed set of action (Slot request) and then serves a goal matching with the obtained information. As mentioned, a dialogue will be successful if and only if the user will get satisfied with the served goal and all its queries will be answered within the maximum dialogue length limit (20). However, the rule based agent is always requesting some slots rather than informing user queries. The DQN variants (Fig 9b) perform better than the AC agent (Fig 9c) because it is a more sample efficient algorithm for a small environment.

Fig 9b, 9d and 9e illustrate how our agents DQN_*TR*_ and DQN_*TR*+*SR*_ agent learns over episodes in terms of *reward, success rate*. In these figures, TR & SR refer to task-oriented reward and sentiment-based reward, respectively. Each episode simulates 100 dialogue. In early training, the DQN_*TR*+ *SR*_ agent’s reward is lesser than DQN_*TR*_ as it gets an additional negative reward (sentiment reward) for each inappropriate action. The VAs learn better with joint reward (TR and SR) rather than any of them alone. Task based reward motivates VA to choose actions that can help in getting more slot information and also to complete the dialogue in less number of turns. Whereas sentiment-based reward provides explicit and accurate feedback about agent behavior such as redundant actions and inappropriate actions. From Fig 9f, it can be observed that the agent learns to complete dialogue conversation in less number of turns over episodes.

The proposed VA is learning to act optimally in the environment through taking appropriate action, including re-result if it finds any goal discrepancy followed by an updated goal in the present dialogue state. In Fig 9d, it can be observed that the DQN_*TR*+*SR*_ agent is getting less and less negative reward over episodes. It implies that the agent is learning to avoid the behavior, which is leading to a penalty either because of negative user sentiment or task failure. The agent is able to recognize goal deviation through dialogue state after some initial training episodes and then learns to re-serve the user with an updated goal rather than ending the dialogue without completion. Once it senses a few successful dialogue trajectories, it heads to the true estimation of the Q value of an action given a dialogue state and hence the underlying state-action distribution Π(*A*|*S*).

Also, we have performed Welch’s T-test [71] for statistical significance measurement. The test is conducted between the DQN_*TR*+*SR*_ model and the remaining models at 5% significance level. The results are reported in Table 8. All the p-values are less than 0.05, which establish that the obtained improvement by the proposed model over baselines are statistically significant.

**Table 8:** Statistical significance test result: p value at 5% significance level.

Model	Success rate	Dialogue Length
AC_*TR*_	1.03e^−08^	1.51e^−08^
AC_*TR*+*SR*_	8.77e^−08^	8.62e^−09^
VDQN_*TR*_	3.59*e*^−10^	3.22*e*^−06^
VDQN_*TR*+*SR*_	3.79*e*^−09^	4.85*e*^−05^
DQN_*TR*_	4.09*e*^−05^	6.77*e*^−03^

### 7.2 Human evaluation

**Fig 10:**
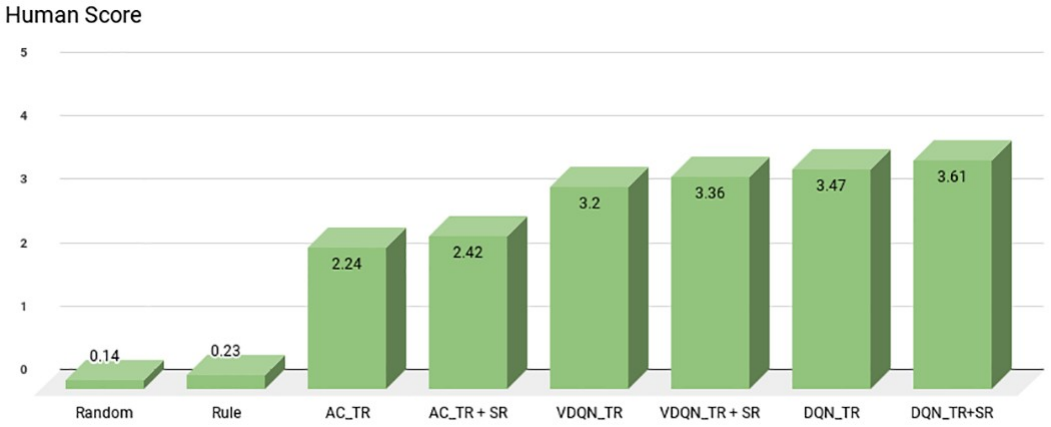
Human score for different baselines and proposed model.

To further quantify the quality of the actions picked up by the dialogue manager, *human evaluation* is also conducted. Two researchers from authors’ affiliation (independent from the authors) were chosen to perform this task. They were asked to score each dialogue during testing in a range of 0 (extremely poor) to 5 (extremely good) depending on the agent’s behavior. 0: incorrect or failed, 1: near to failure, 2: neutral, 3: average, 4: good, 5: extremely good. Final results reported are calculated on a random subset of 100 test dialogues as follows:

$$\begin{array}{c} HS=(\sum_{n=1}^{n=100}s_{i})/100 \end{array}$$ (19)

where *s*_*i*_ is the score [0, 5] assigned to $s_{i}^{th}$ test dialogue based on agent actions which were chosen throughout the dialogue conversation, *s*_*i*_. The values of human evaluation metric for different models are reported in Fig 10. We computed the Fleiss’ kappa [72] for the above metrics to measure inter-rater consistency. A value of 0.73 is obtained that shows significant mutual agreement.

**Fig 11:**
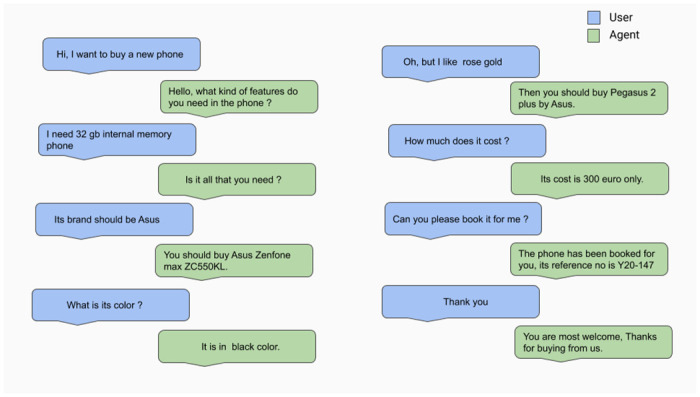
VA performance during testing—sample1.

**Fig 12:**
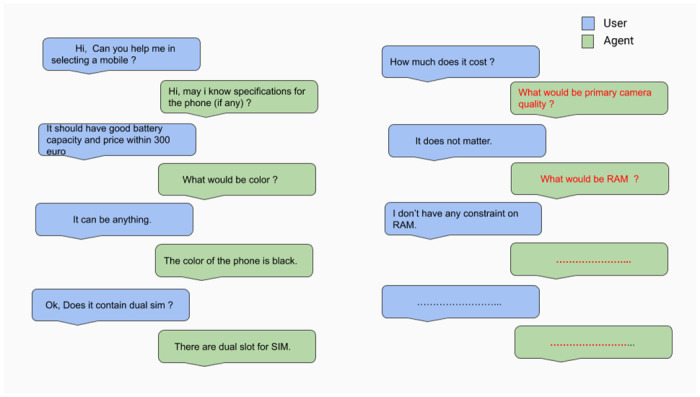
VA performance during testing—sample2.

Conversations reported in Fig 11 illustrate how the user changes his/her mind/goal after knowing some information (primary camera quality) about shown result, and the VA is able to handle it successfully. Though the agent is trained with both sentiment and task oriented reward functions, there are still a few cases where the agent fails because of some unnecessary informs and queries, as shown in Fig 12.

### 7.3 Error analysis

**Fig 13:**
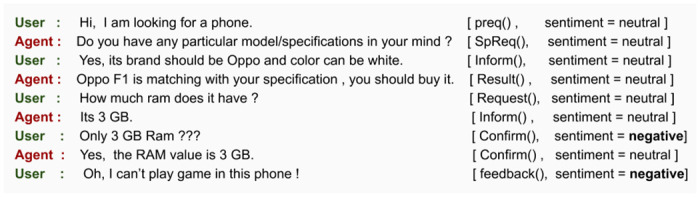
A sample that shows new goal formulation error.

A detailed error analysis leads to the following observations:

- **New goal formulation error**: A few times, the VA fails to recognize a user’s new goal when they don’t express their feedback implicitly. In Fig 13, the VA was not able to understand deviation/user feedback due to its complication. It is difficult for any VA to process such complex situations.
- **Intent classification error**: Sometimes, the incorporated NLU system misclassifies user intent that leads to an inappropriate action selection by the proposed DM. One such observed example is as follows: User— “*Please book the phone if it is available in black*”, Classified Intent—done. Here the dialogue ended without the user’s desired task completion.
- **Slot filling error**: In a task-oriented dialogue system, slot-value pairs are crucial as they constitute the task goal. We observed two kinds of slot errors: missing slot and incorrect/incomplete slot value.
  1. **Missing slot**: User— “*I need a phone with both 13 PM primary camera and secondary camera*”. Tagged Slot Sequence: O O O O O O P-Camera O O O O O O O. Although user informed about both primary camera and secondary camera, the predicted slot is “P-Camera = 13”.
  2. **Incorrect/Incomplete slot value**: User— “*I need a phone in light blue color*”. Tagged Slot Sequence: O O O O O O B-Color O O i.e., the predicted slot is “Color = blue” but it should be “Color = light blue”.

### 7.4 Comparison with the state of the art

To the best of our knowledge, this is the first effort towards developing a dynamic goal adapted virtual agent. It might not be entirely fair to compare our VA with some traditional task oriented dialogue agents as they have not been trained for such scenarios. Still, to establish the importance and efficacy of the proposed system, we experimented with a few recent task-oriented dialogue systems [17, 20, 30, 41] for the proposed problem. n [17], authors proposed a simple yet effective methodology for optimizing dialogue policy using reinforcement learning. With their reward model [17], the agent completely flatters, so we shaped the reward model. In addition to the mentioned immediate reward, the agent gets a reward of +1 and -1 when it succeeds and fails, respectively. In our case, numSlots2Confirm = 2, DL = -0.05. In [41], the authors proposed a Natural Language Understanding (NLU) robust Goal Oriented Bot (GO-Bot) for movie ticket booking. In [30], the authors presented a Sentiment aware Virtual agent (SentiVA) that establishes the importance of immediate sentiment-based reward in a multi-intent dialogue setting using Hierarchical Reinforcement Learning (HRL). These models’ performances on the proposed problem are reported in Tables 9–11.

**Table 9:** Performance of the SimpleDS for dynamic goal setting.

Evaluation Metric	Result
Avg Episodic Reward	-21.16
Success rate	0.003
Avg. Dialogue length	8.82

**Table 10:** GO-Bot performance for dynamic goal setting scenarios.

Evaluation Metrics	Result
Avg. Episodic Reward	-34.90
Success rate	0.001
Avg. Dialogue Length	14.96

**Table 11:** SentiVA’s performance for dynamic goal setting scenarios.

Evaluation Metrics	Result
Avg. Reward	-0.73
Success rate	0.003
Avg. Dialogue Length	15.12

These models do not converge at all as they fail to sense successful dialogue trajectories. Although a user expresses his/her feedback/update, these VAs fail to incorporate and update the goal; thus, leading to unsuccessful dialogue termination. Their immediate reward turns out to be insignificant in cumulative reward Q(S, a) update compared to the huge penalty provided for each unsuccessful termination. It becomes difficult for these VAs to distinguish inappropriate action from the appropriate one. Henceforth, they got stuck at a local maximum. We also found a lot of action repetition and inappropriate behavior of SimpleDS agent, which may be due to a trivial and sparse reward model. There are very few cases where the agent’s initial served goal is already aligned with the user updated goal component coincidentally, which leads to successful dialogue termination.

It is very hard to ensure an optimal dialogue policy without a huge training corpus in a supervised setting. Even though it is trained with a large corpus, a small deviation in the language dictionary may significantly deteriorate the VA performance. On the other hand, RL is proven to be the state of the art for decision-making problems such as dialogue management, even with significant small gold data. An RL agent senses the importance of each action as well as action sequences by exploring infinitely large state-action space with the help of a user simulator that mimics end-user behaviors. Sometimes, it becomes hard to train an RL agent as it requires significant human involvement for reward model tuning. Once a reward model is tuned, the agent can be trained easily, and also, the setup can be utilized for similar problems/other domains with minimal change.

### 7.5 Supervised setting

We also investigated a supervised approach for the proposed problem, i.e., a Seq2Seq model, TSCP [20] that uses a single seq2seq model with a CopyNet mechanism. The model has been evaluated on two different domains, namely restaurant and calendar with the primary task of restaurant reservation and calendar scheduling, respectively. Though it is not entirely fair to compare RL based dialogue agent with a Seq2Seq chatbot because of different requirements and evaluation space, we can steadily equate them in terms of the primary objective, i.e., task success. Hence, we computed evaluation metrics (Success rate and Dialogue length) in addition to the metrics utilized for evaluation of the TSCP model. The evaluation metric, entity match rate, indicates the model’s language understanding capability, i.e., whether the system can predict the correct entity expressed by a user. Success F1 indicates F1 score of user requested slots during a conversation. The obtained result have been reported in Table 12.

**Table 12:** TSCP model performance for dynamic goal setting scenarios.

Model	Success rate	Avg Dialogue Length	Entity match rate	Success F1	BLEU
TSCP	0.56	8.96	0.701	0.842	0.274

The model performs poorly to predict correct user entities, which is a significant factor for unsuccessful dialogue. The number of slots and distinct slot values of our knowledge base are comparatively higher, which makes the entity’s prediction task harder. In some cases, the agent keeps on serving the initial goal even though the user shows negative sentiment towards the served goal, which leads to unsuccessful dialogue. Also, sometimes the agent fails to inform the user requested slot with an appropriate value.

## 8 Conclusion and future work

This paper presents the first step towards developing a dynamic goal oriented virtual agent which is capable of handling the variations in user goal in real-time. The variation in goal can arise because a user may want to decide his/her goal depending upon the determined goal components and VA serving capability or due to a mismatch in the implicit slot values of the user. We have proposed an end to end dynamic goal driven virtual agent by incorporating GDM module with a reinforcement learning based dialogue manager. It is an interactive VA that utilizes task-specific reward and sentiment-based reward to deal with a dynamic goal. Also, we created a data set named *DevVA* which contains dialogues between users and the agent; samples of this dataset are annotated with intent, slot and sentiment. The dataset will be made publicly available for accelerating research towards dynamic goal driven dialogue system. The results show that the developed VA is capable of handling dynamic goals with a significantly high success rate and user gratification (human score) in a reasonable number of dialogue turns.

The proposed system can be useful for any task oriented dialogue system where the end-users determine their goal dynamically. It enhances the capability of a typical VA to deal with a more practical scenario with ease. As the system is trained primarily with a user simulator, it can be applied to other domains with minimal changes.

### 8.1 Limitations

Despite impressive results, the VA lacks in these two aspects currently that make it generalized and less alluring. *i. Intensifier Resolution*: Some heuristic rules have been used for quantifying intensifiers. This should be replaced by some automated value determined based on the user’s personalization/gender/profession. *ii. Template-based NLG module*: The NLG module of the proposed end to end system is retrieval-based. So the response presented to end users may not be appealing despite of appropriate VA action.

### 8.2 Future works and recommendations

In future, a persuasion module can be integrated with the DM to enable the VA in persuading the users for a similar goal if the proposed goal is out of the scope of the VA’s serving capability. Sometimes users do not express their sentiments explicitly, which might be a challenge for identifying goal deviation. Context-aware sentiment is an interesting approach to be explored to deal with this anomaly. A persona can contribute significantly in making the VA more user adapted and personalized. Sometimes users prefer to show an image rather than explaining in text. It becomes hard to describe a phone’s few aspects such as style and color through text; hence, multi-modality will surely improve user easefulness. All these aspects will be addressed in the future.
